# Gastric Bezoar: Cause of Weight Loss in a Patient With Previous Bariatric Surgery

**DOI:** 10.7759/cureus.20139

**Published:** 2021-12-03

**Authors:** Ahmed Alemam, Dongmin Shin, Bhavna Balar

**Affiliations:** 1 Gastroenterology, BronxCare Health System, Bronx, USA; 2 Internal Medicine, BronxCare Health System, Bronx, USA

**Keywords:** gastric bezoar, gastroenterology and endoscopy, adult gastroenterology, gastroenterology

## Abstract

Gastric bezoar is a concretion of undigested material found in the stomach and is classified by its composition. Patients may remain asymptomatic or present with a variety of gastrointestinal symptoms. Upper gastrointestinal endoscopy is required to establish the diagnosis. Treatment options include chemical dissolution, endoscopic removal, or surgical removal. Here, we present a rare case of gastric bezoar in a patient with a remote history of bariatric surgery presenting with acute weight loss.

## Introduction

A bezoar is a conglomerate of indigestible foreign bodies or undigested materials often found as a hard mass that may exist anywhere along the gastrointestinal tract, most commonly in the stomach. Although it varies among studies, the prevalence of a bezoar is around 0.4%. The prevalence may be higher in certain population groups as food culture plays a big role (e.g., consumption of persimmons) [1]. Patients at risk of developing gastric bezoars are those with various psychiatric disorders such as trichotillomania and trichotillophagia, gastrointestinal tract anatomic abnormalities. Other risk factors include ingestion of bulk-forming agents or extended-release medications, mastication disorders, dehydration, vegetarian diet, and pica consumption. Common medical conditions associated with gastric bezoars include diabetes mellitus, hypothyroidism, gastric dysmotility, gastrointestinal amyloidosis, and scleroderma [2-4]. Patients with a history of gastric surgery such as pyloroplasty with vagotomy and bariatric surgery are also at risk for gastric bezoar formation due to subsequent postoperative changes like decreased peristalsis, low gastric acid environment, abnormal pyloric function, or gastric outlet obstruction [5-8]. Here, we present a case of gastric bezoar discovered during upper endoscopy done to evaluate acute, unexpected weight loss in a patient who had bariatric surgery 25 years ago.

## Case presentation

A 68-year-old Hispanic woman initially presented to the clinic with complaints of heartburn, regurgitation of food, decreased appetite, intermittent dysphagia, and occasional vomiting for three months. She also reported unintentional weight loss of 8 kilograms over a similar duration. Patient reported having occasional constipation but denied diarrhea, hematochezia, melena, dizziness, fatigue, shortness of breath, or palpitations. Patient has been taking omeprazole without much relief of her symptoms. Patient’s medical history was significant for hypothyroidism, major depressive disorder, hypertension, prediabetes, dyslipidemia, gastroesophageal reflux disease (GERD), and chronic obstructive pulmonary disease (COPD). Patient had a vertical banded gastroplasty and Nissen fundoplication done 25 years ago. Patient denied any family history of gastrointestinal disease or malignancy. Patient had a screening colonoscopy done 14 years ago which showed one tubular adenoma and again three years ago which only showed diverticulosis and hemorrhoids. Social history was significant for a prior history of smoking (40 pack-years) and occasional marijuana use for nausea. Physical examination was unremarkable. Laboratory test results are included in Table 1.

**Table 1:** Laboratory test results HDL: high-density lipoprotein; LDL: low-density lipoprotein; T3: triiodothyronine

Labs	Value	Reference range
White blood cell	7.5	4.8-10.8 K/µL
Hemoglobin	12.4	12.0-16.0 g/dL
Hematocrit	37.9	42.0-51.0%
Platelet	239.0	150-400 K/µL
Sodium	137.0	135-145 mEq/L
Potassium	4.2	3.5-5.0 mEq/L
Chloride	101.0	98-108 mEq/L
Bicarbonate	29.0	24-30 mEq/L
Glucose	114.0	70-120 mg/dL
Blood urea nitrogen	13.0	6-20 mg/dL
Creatinine	0.7	0.5-1.5 mg/dL
Calcium	9.6	8.5-10.5 mg/dL
Total protein	7.0	5.8-8.3 g/dL
Albumin	4.3	3.2-4.6 g/dL
Bilirubin, total	0.2	0.2-1.1 mg/dL
Alkaline phosphatase	83.0	43-160 unit/L
Aspartate transaminase	15.0	9-36 unit/L
Alanine aminotransferase	12.0	5-40 unit/L
Cholesterol	214.0	170-240 mg/dL
HDL cholesterol	63.0	34-82 mg/dL
LDL cholesterol	137.0	≤ 160 mg/dL
Triglycerides	68.0	60-150 mg/dL
Thyroid stimulating hormone	0.19	0.40-4.50 mIU/L
Free thyroxine	1.34	0.80-2.00 ng/dL
T3	99.0	60-181 ng/dL
Hemoglobin A1c	5.8	4.7-6.4%

**Figure 1:**
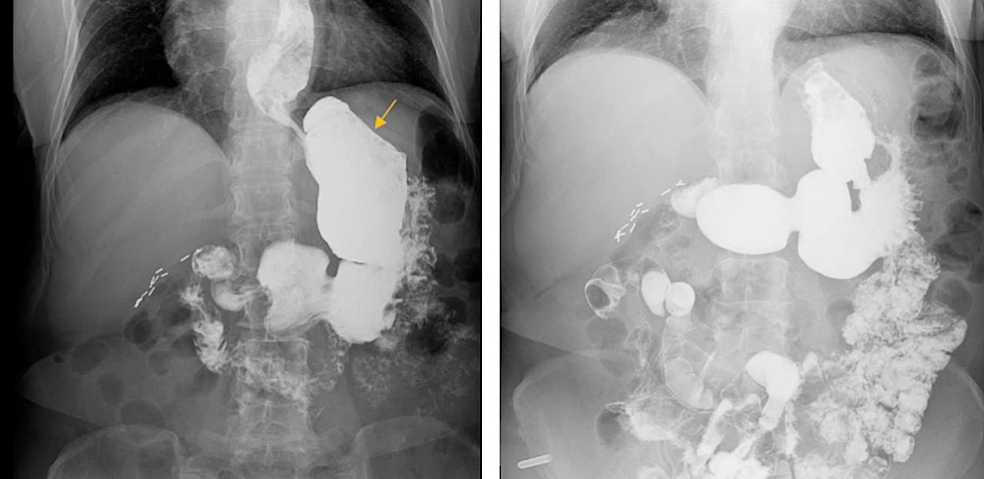
Upper GI series Contrast is seen entering the gastric pouch (arrow) and the rest of the stomach.

Upper GI series was done and the contrast was initially seen entering the gastric pouch, then to the rest of the stomach which were findings consistent with the patient's vertical banded gastroplasty history (Figure 1). The decision was made to proceed with esophagogastroduodenoscopy (EGD) in view of unexpected weight loss.

**Figure 2:**
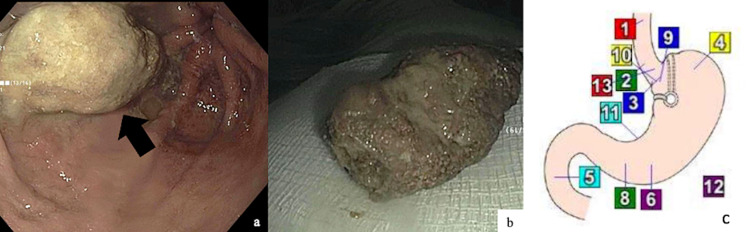
Endoscopic findings Gastric bezoar found in the gastric pouch (a) and after removal (b). Anatomic diagram of the stomach after vertical banded gastroplasty (c).

Endoscopic examination revealed evidence of patent vertical banded gastroplasty with a normal-sized gastric pouch containing a phytobezoar, which was the likely cause of the intermittent obstruction and most of the symptoms that the patient was experiencing (Figures 2a-2c). The phytobezoar was approximately 20 mm tall in size and was successfully removed with a Roth Net (Mentor, OH: STERIS). Rest of the endoscopic findings were unremarkable, the scope passed through the gastric pouch easily, leading to normal antrum and pylorus. Upon follow-up visit, the patient reported resolution of her previously reported symptoms and returned back to her previous weight.

## Discussion

Bezoars can be asymptomatic and be found incidentally in patients undergoing esophagogastroduodenoscopy (EGD), or present with a variety of gastrointestinal symptoms including abdominal pain, anorexia, nausea, vomiting, early satiety, or gastrointestinal bleeding. Symptoms related to gastrointestinal bleeding such as hematemesis, hematochezia, melena, and anemia, are related to the development of gastric ulcer(s) due to pressure necrosis caused by the bezoar [9]. Rarely, gastric bezoars can also cause gastric outlet obstruction or it can migrate to the small intestine and present as small bowel obstruction [9-11].

Bezoars are classified according to their composition. Phytobezoars, the most common type of bezoars, are composed of vegetable matter. Diospyrobezor, a subtype of phytobezoar, is composed of persimmon fruit. It is harder in consistency compared to other phytobezoars and can be resistant to dissolution treatment and thus accounts for the majority of cases [12]. Trichobezoars are composed of hair, which are rare and often diagnosed in those with psychiatric comorbidities [13]. Pharmacobezoars are caused by various ingested medications, especially the extended-release drugs coated with a chemical compound derived from the plant substance cellulose, or enteric-coated drugs that remain insoluble in the stomach [9,14,15]. Lactobezoar is an undigested mass composed of milk and mucus components affecting milk-fed infants. Its incidence decreased dramatically in recent years due to improvement in synthetic milk composition and advances in premature newborn management [9,16,17].

Upper gastrointestinal endoscopy is the most important modality in the diagnosis and management of a gastric bezoar. Endoscopically, a gastric bezoar appears as a single or multiple ball-like masses in the gastric fundus or antrum. The color can range from beige to yellow, green, dark brown, or black depending on the composition of the bezoar [9,18]. CT scan can be helpful in detecting small bowel bezoars along the GI tract that require surgical intervention [19,20].

Treatment options for gastric bezoar include chemical dissolution, endoscopic removal, or surgical removal. Chemical dissolution is done by administering chemical agents via gastric lavage, ingestion, or by direct endoscopic injection. Among the chemical agents, Coca-Cola has been shown to be highly effective either as a single therapy or combined with endoscopic intervention [12]. Endoscopic treatment involves fragmentation and removal of the bezoar using various devices such as net devices, forceps, snare, a basket catheter, electrohydraulic lithotripsy device [18]. Surgical removal is reserved for those who fail chemical dissolution and endoscopic treatment or for those with complications such as obstruction or perforation. Traditionally bezoars were removed by laparotomy, but laparoscopic removal is growing as the surgical intervention of choice [20].

## Conclusions

Gastric bezoar is an uncommon condition that may be found incidentally during endoscopy or present with various gastrointestinal symptoms. A clinician should consider it as a differential diagnosis especially in those who are at higher risk, such as those with delayed gastric emptying, history of gastric surgery, high consumption of certain food including persimmon fruit, and psychiatric patients who ingest hair or other foreign materials. Endoscopic investigation and retrieval of the bezoar will confirm the diagnosis and successfully treat the condition in most cases.
